# Artificial neural network model to predict post-hepatectomy early recurrence of hepatocellular carcinoma without macroscopic vascular invasion

**DOI:** 10.1186/s12885-021-07969-4

**Published:** 2021-03-16

**Authors:** Rong-yun Mai, Jie Zeng, Wei-da Meng, Hua-ze Lu, Rong Liang, Yan Lin, Guo-bin Wu, Le-qun Li, Liang Ma, Jia-zhou Ye, Tao Bai

**Affiliations:** 1 Department of Hepatobilliary & Pancreatic Surgery, Guangxi Medical University Cancer Hospital, 71 He Di Road, Nanning, China; 2 Department of Experimental Research, Guangxi Medical University Cancer Hospital, Nanning, 530021 China; 3 Guangxi Liver Cancer Diagnosis and Treatment Engineering and Technology Research Center, Nanning, 530021 China; 4 Department of First Chemotherapy, Guangxi Medical University Cancer Hospital, Nanning, 530021 China

**Keywords:** Hepatocellular carcinoma, Curative hepatectomy, Early recurrence, Prognostic factors, Artificial neural network

## Abstract

**Background:**

The accurate prediction of post-hepatectomy early recurrence (PHER) of hepatocellular carcinoma (HCC) is vital in determining postoperative adjuvant treatment and monitoring. This study aimed to develop and validate an artificial neural network (ANN) model to predict PHER in HCC patients without macroscopic vascular invasion.

**Methods:**

Nine hundred and three patients who underwent curative liver resection for HCC participated in this study. They were randomly divided into derivation (*n* = 679) and validation (*n* = 224) cohorts. The ANN model was developed in the derivation cohort and subsequently verified in the validation cohort.

**Results:**

PHER morbidity in the derivation and validation cohorts was 34.8 and 39.2%, respectively. A multivariable analysis revealed that hepatitis B virus deoxyribonucleic acid load, γ-glutamyl transpeptidase level, α-fetoprotein level, tumor size, tumor differentiation, microvascular invasion, satellite nodules, and blood loss were significantly associated with PHER. These factors were incorporated into an ANN model, which displayed greater discriminatory abilities than a Cox’s proportional hazards model, preexisting recurrence models, and commonly used staging systems for predicting PHER. The recurrence-free survival curves were significantly different between patients that had been stratified into two risk groups.

**Conclusion:**

When compared to other models and staging systems, the ANN model has a significant advantage in predicting PHER for HCC patients without macroscopic vascular invasion.

**Supplementary Information:**

The online version contains supplementary material available at 10.1186/s12885-021-07969-4.

## Background

Hepatocellular carcinoma (HCC) is the sixth most common form of malignancy and the fourth leading cause of cancer deaths worldwide [1]. The most common curative treatment for early HCC patients is hepatectomy [2–4]. However, its effectiveness is limited by the high incidence of tumor recurrence in the postoperative period (up to 60%), leading to poor long-term survival in HCC patients [2–4]. Therefore, accurate prognostic prediction of postoperative tumor recurrence is consequential in the screening and choice of adjuvant therapies for high-risk patients.

Post-hepatectomy tumor recurrence can be divided into early or late recurrence using 2 y as the cut-off point [5–9]. Numerous studies have reported that post-hepatectomy early recurrence (PHER) is associated with intrahepatic metastases from primary tumors that cannot be clinically detected, while late recurrence results from tumor formation following liver cirrhosis [10]. Patients with PHER typically have worse long-term survival prognoses than those with late recurrence [11]. PHER is key in the poor prognosis of HCC patients following curative hepatectomy. Therefore, the establishment of an accurate, reliable, and specific PHER prediction model may provide a reliable means for choosing postoperative adjuvant treatments in high-risk patients, such as radiofrequency ablation (RFA), transcatheter arterial chemoembolization (TACE), or sorafenib.

Artificial neural network (ANN) models are established mathematical tools that mimic the features of neurons. They have data distributions with large-scale parallel structures and processing mechanisms similar to the biological brain [12]. ANN models have been used to process information in extremely complicated biological systems containing multiple related factors [13, 14]. Recently, ANN models have been used in the prognostic assessment of various cancers [15–17]. However, few studies on its application in HCC have been published. This study aimed to develop an ANN model to assess PHER risk of HCC patients without macroscopic vascular invasion who underwent hepatectomy. Its predictive ability was compared with a Cox proportional hazards (CPH) model, several preexisting recurrence models, and commonly used staging systems.

## Methods

### Patients

Data from HCC patients who underwent liver resection from September 2013 to December 2019 in our hospital were enrolled in this study. The inclusion criteria were: preoperative Child-Pugh score of A or B, underwent a curative liver resection, and HCC diagnosis from post-operative pathology. The exclusion criteria were: had received TACE, RFA, or systemic treatment; tumor invasion in main hepatic veins, portal veins, or adjacent organs; hospital mortality after liver resection; and incomplete clinical data. The 903 HCC patients who met the criteria were randomly classified into derivation (*n* = 679) and validation cohorts (*n* = 224) in a ratio of 3:1 (Supplementary Fig. 1). This study was approved by the Ethical Committee of the Guangxi Medical University Cancer Hospital and was performed in compliance with the Helsinki Declaration. Written informed consent was obtained from all patients.

### Commonly used clinical staging systems

Patients were staged using the following systems: Barcelona Clinic Liver Cancer [18], TNM (American Joint Committee on Cancer, 8th edition) [19], Okuda [20], China Liver Cancer (CNLC) [21], Hong Kong Liver Cancer [22], French [23], Cancer of the Liver Italian Program [24], and Japan Integrated Staging [25].

### Surgical procedures and follow-ups

Liver resection was performed when preoperative imaging indicated that all tumors can be resected within the hepatic functional reserve. Major hepatectomy was defined as a resection of ≥ three Couinaud’s segments [26]. Additional details and indications for liver resection are described in a previous study [27].

All patients were followed-up every two to 3 months until death or withdrawal. Routine follow-up included the determination of laboratory parameters, abdominal ultrasounds, and computed tomography (CT) or magnetic resonance imaging (MRI). All serological parameters were assayed and analyzed in the early morning of the first day post-admission. Depending on liver functional reserve, general health status, and disease extent, patients with recurrent tumors underwent re-resection or treatment with RFA, TACE, or sorafenib [28]. HCC recurrence or metastasis was determined based on CT and/or MRI results, regardless of whether serum α-fetoprotein (AFP) levels were elevated [29]. HCC recurrence was defined as the imaging of new lesions in the residual hepatic tissue or the occurrence of distant metastases. We defined PHER as within 2 y post-surgery from the date of the hepatectomy to the date of the first diagnosis of HCC recurrence [5].

### Development of the CPH and ANN models

The CPH and ANN models were established based on the identification of significant PHER prognostic factors using univariable and multivariable Cox analyses. The CPH model is similar to multiple linear regression in that it explores the relationship between a hazard and its associated independent explanatory factors over a period of time. It describes the impact of risk factors on a patient’s treatment using a parameter called the risk ratio [30]. Here, the CPH model used the sum of the relevant risks affecting the hazard function to predict PHER risk in HCC patients without macroscopic vascular invasion.

The ANN model was established via a multilayer perceptron network (MLP), which is a popular architecture and a new layer feed-forward neural network design tool consisting of input nodes, hidden layers, and an output node [13]. MLP models are always trained using back-propagation algorithms. When data is provided to neural groups through the input layers, the first-layer neurons propagate the weighted data and randomly select the biases using the hidden layers. When the net sum at the hidden nodes is confirmed, the transfer function can then be used to provide output responses at the nodes [16, 17].

In this study, eight prognostic indicators (hepatitis B virus deoxyribonucleic acid [HBV-DNA] load, γ-glutamyl transpeptidase [GGT] levels, AFP levels, tumor size, tumor differentiation, microvascular invasion [MVI], satellite nodules, and blood loss) were selected as the input nodes, and one indicator (with and without PHER) was used as the output node. Details of the ANN model are described in a previous study [14]. To avoid over-optimization, the results reported in this study were the optimal results following repeated randomized trials.

### Statistical analysis

We randomly divided patients into derivation and validation cohorts, as detailed above (see section Patients). The cut-offs values for the included parameters were confirmed using Youden’s index (i.e., sensitivity + specificity − 1) and other published reports [27, 29]. These categorical variables are presented as frequencies and percentages and then compared using a chi-squared test. Recurrence-free survival (RFS) curves were assessed via the Kaplan-Meier method and compared using the log-rank test. The predictive abilities of the ANN, CPH, some preexisting recurrence models, as well as commonly used clinical staging systems were calculated using the areas under the receiver-operating characteristic (ROC) curves (AUCs) and decision curve analyses (DCA) [31]. Calibration plots were applied to test the calibration capacity of the ANN model. For its clinical application, Youden’s index was calculated to determine the optimal cut-off value for predicting PHER. All patients were then classified as high-risk and low-risk groups.

All statistical analyses were performed using SPSS (v25.0). All statistical tests were two-tailed, and *P* values < 0.05 were considered statistically significant.

## Results

### Baseline characteristics

A total of 903 HCC patients who had received curative liver resection were included in this study. The cohort consisted of 772 males and 131 females. Despite all patients having preserved hepatic function, 760 (84.2%) were infected with the hepatitis B virus, and 392 (43.4%) had HBV-DNA loads > 10^4^ IU/mL.

With respect to tumor status, 486 patients (53.8%) had tumor sizes > 5 cm, and 191 patients (21.2%) had multiple tumors. More specifically, 36.7% of patients had MVI, 8.5% had satellite nodules, 52.4% had tumor necrosis, 46.5% had poorer tumor differentiation, and 44.6% had cirrhosis. Details of the HCC staging systems are listed in Supplementary Table 1.

Regarding the surgeries, 361 patients (40.0%) underwent major hepatectomy, 244 patients (27.0%) suffered from blood loss > 400 mL, 99 patients (11.0%) received blood transfusions, and 86 patients (9.5%) presented with resection margins > 1 cm.

Baseline characteristics are presented in Table 1; they did not significantly differ between the derivation and validation cohorts (*P* > 0.05 for all comparisons).

**Table 1:** Patient demographics and tumor characteristics of whole cohort, derivation whole cohort, derivation cohort and validation cohort

Variables	Whole cohort (*n* = 903)	Derivation cohort (*n* = 679)	Validation cohort (*n* = 224)	*P* value
Age, years				0.869
> 60	270 (29.9)	204 (30.0)	66 (29.5)	
≤ 60	633 (70.1)	475 (70.0)	158 (70.5)	
Sex				0.743
Male	772 (85.5)	579 (85.3)	193 (86.2)	
Female	131 (14.5)	100 (14.7)	31 (13.8)	
HBsAg				0.339
Positive	760 (84.2)	576 (84.8)	184 (82.1)	
Negative	143 (15.8)	103 (15.2)	40 (17.9)	
HBeAg				0.522
Positive	255 (28.2)	188 (27.7)	67 (29.9)	
Negative	648 (71.8)	491 (72.3)	157 (70.1)	
HBV-DNA, IU/mL				0.847
> 10^4^	392 (43.4)	296 (43.6)	96 (42.9)	
≤ 10^4^	511 (56.6)	383 (56.4)	128 (57.1)	
Antiviral therapy				0.274
Yes	431 (47.7)	317 (46.7)	114 (50.9)	
No	472 (52.3)	362 (53.3)	110 (49.1)	
PT, s				0.180
> 13	333 (36.9)	242 (35.6)	91 (40.6)	
≤ 13	570 (63.1)	437 (64.4)	133 (59.4)	
T-Bil, μmol/L				0.580
> 17.1	261 (28.9)	193 (28.4)	68 (30.4)	
≤ 17.1	642 (71.1)	486 (71.6)	156 (69.6)	
ALB, g/L				0.152
> 40	351 (38.9)	273 (40.2)	78 (34.8)	
≤ 40	552 (61.1)	406 (59.8)	146 (65.2)	
ALT, U/L				0.324
> 40	318 (35.2)	233 (34.3)	85 (37.9)	
≤ 40	585 (64.8)	446 (65.7)	139 (62.1)	
AST, U/L				0.119
> 40	383 (42.4)	278 (40.9)	105 (46.9)	
≤ 40	520 (57.6)	401 (59.1)	119 (53.1)	
GGT, U/L				0.509
> 60	350 (38.8)	259 (38.1)	91 (40.6)	
≤ 60	553 (61.2)	420 (61.9)	133 (59.4)	
Ascites				0.719
Yes	95 (10.5)	70 (10.3)	25 (11.2)	
No	808 (89.5)	609 (89.7)	199 (88.8)	
Child-Pugh grade				0.793
A	839 (92.9)	630 (92.8)	209 (93.3)	
B	64 (7.1)	49 (7.2)	15 (6.7)	
AFP, ng/mL				0.218
> 400	312 (34.6)	227 (33.4)	85 (37.9)	
≤ 400	591 (65.4)	452 (66.6)	139 (62.1)	
CSPH				0.388
Yes	95 (10.5)	68 (10.0)	27 (12.1)	
No	808 (89.5)	611 (90.0)	197 (87.9)	
Tumour size, cm				0.054
> 5	486 (53.8)	353 (52.0)	133 (59.4)	
≤ 5	417 (46.2)	326 (48.0)	91 (40.6)	
Tumour number				0.114
Multiple	191 (21.2)	152 (22.4)	39 (17.4)	
Single	712 (78.8)	527 (77.6)	185 (82.6)	
Cirrhosis				0.760
Yes	403 (44.6)	305 (44.9)	98 (43.8)	
No	500 (55.4)	374 (55.1)	126 (56.3)	
Tumor differentiation				0.060
Grade III or IV	420 (46.5)	328 (48.3)	92 (41.1)	
Grade I or II	483 (53.5)	351 (51.7)	132 (58.9)	
Tumor encapsulation				0.678
Complete	485 (53.7)	362 (53.3)	123 (54.9)	
None/incomplete	418 (46.3)	317 (46.7)	101 (45.1)	
Microvascular invasion				0.195
Yes	331 (36.7)	257 (37.8)	74 (33.0)	
No	572 (63.3)	422 (62.2)	150 (67.0)	
Satellite nodules				0.600
Yes	77 (8.5)	56 (8.2)	21 (9.4)	
No	826 (91.5)	623 (91.8)	203 (90.6)	
Necrosis				0.959
Yes	473 (52.4)	356 (52.4)	117 (52.2)	
No	430 (47.6)	323 (47.6)	107 (47.8)	
Resection margin, cm				0.816
> 1	86 (9.5)	64 (9.4)	22 (9.8)	
≤ 1	817 (90.5)	615 (90.6)	202 (90.2)	
Operation time, min				0.384
> 200	365 (40.4)	280 (41.2)	85 (37.9)	
≤ 200	538 (59.6)	399 (58.8)	139 (62.1)	
Blood loss, mL				0.661
> 400	244 (27.0)	186 (27.4)	58 (25.9)	
≤ 400	659 (73.0)	493 (72.6)	166 (74.1)	
Blood transfusion				0.380
Yes	99 (11.0)	78 (11.5)	21 (9.4)	
No	804 (89.0)	601 (88.5)	203 (90.6)	
Extent of resection				0.391
Major resection	361 (40.0)	266 (39.2)	95 (42.4)	
Minor resection	542 (60.0)	413 (60.8)	129 (57.6)	

*Abbreviations*: *HBsAg* hepatitis B surface antigen, *HBeAg* hepatitis Be antigen, *HBV-DNA* hepatitis B virus DNA load, *PT* prothrombin time, *T-Bil* total bilirubin, *ALB* albumin, *ALT* alanine transaminase, *AST* aspartic aminotransferase, *GGT* γ-glutamyl transpeptadase, *AFP* α-fetoprotein, *CSPH* clinically significant portal hypertension

### PHER

Of all the patients, 324 (35.9%) had PHER (intra-hepatic recurrence: *n* = 280, extra-hepatic recurrence: *n* = 20, concurrent intra- and extra-hepatic recurrence: *n* = 24). The mean PHER duration was 16.9 months (95% confidence interval [CI]: 16.2–17.5 months). The PHER rate between the derivation (236/679, 34.8%) and validation (88/224, 39.2%, *P* = 0.220) cohorts did not significantly differ.

### Identification of independent prognostic factors

The univariable Cox analysis revealed that hepatitis B surface antigen level, HBV-DNA load, antiviral therapy, albumin level, aspartate aminotransferase level, GGT level, prothrombin time, Child-Pugh grade, AFP level, tumor size, tumor differentiation, MVI, satellite nodules, surgical time, blood loss, blood transfusion, and major resection were all related to PHER (Table 2, *P* < 0.05 for all comparisons). Accordingly, these variables were selected for the multivariable model analysis. The Cox multivariable analysis indicated that HBV-DNA, GGT level, AFP level, tumor size, tumor differentiation, MVI, satellite nodules, and blood loss were significantly associated with PHER (Table 2). The RFS curves of these eight prognostic factors are shown in Supplementary Fig. 2.

**Table 2:** Univariate and multivariate analyses of prognostic factors affecting post-hepatectomy early recurrence in the derivation cohort

Variables	Univariate Cox regression			Multivariate Cox regression		
	β	HR (95CI%)	*P value*	β	HR (95CI%)	*P* value
Age > 60 year	−0.289	0.749 (0.558, 1.005)	0.054			
Sex, Male	0.226	1.254 (0.855, 1.840)	0.247			
Positive HBsAg	0.524	1.688 (1.107, 2.575)	0.015	0.286	1.331 (0.839, 2.110)	0.225
Positive HBeAg	0.246	1.278 (0.970, 1.685)	0.081			
HBV-DNA > 10^4^ IU/mL	0.540	1.717 (1.329, 2.218)	< 0.001	0.435	1.544 (1.188, 2.008)	0.001
Antiviral therapy	0.313	1.368 (1.059, 1.767)	0.017	0.068	1.071 (0.800, 1.433)	0.645
PT > 13 s	0.320	1.378 (1.063, 1.786)	0.016	0.179	1.196 (0.911, 1.570)	0.197
T-Bil > 17.1 μmol/L	0.210	1.234 (0.938, 1.623)	0.132			
ALB > 40 g/L	−0.427	0.652 (0.499, 0.853)	0.002	−0.147	0.863 (0.639, 1.165)	0.336
ALT > 40 U/L	0.123	1.131 (0.866, 1.476)	0.366			
AST > 40 U/L	0.588	1.801 (1.394, 2.325)	< 0.001	0.111	1.118 (0.835, 1.496)	0.445
GGT > 60 U/L	0.580	1.785 (1.383, 2.305)	< 0.001	0.388	1.474 (1.131, 1.920)	0.004
Ascites	0.355	1.427 (0.983, 2.071)	0.062			
Child-Pugh grade	0.508	1.662 (1.071, 2.579)	0.024	0.076	1.079 (0.682, 1.707)	0.745
AFP > 400 ng/mL	0.591	1.806 (1.396, 2.337)	< 0.001	0.422	1.525 (1.170, 1.987)	0.002
CSPH	0.224	1.251 (0.838, 1.868)	0.273			
Tumor size > 5 cm	0.816	2.262 (1.719, 2.976)	< 0.001	0.456	1.578 (1.172, 2.214)	0.003
Multiple number	−0.095	0.910 (0.661, 1.253)	0.562			
Cirrhosis	0.231	1.260 (0.976, 1.626)	0.076			
Tumor differentiation (grade III / IV)	0.631	1.880 (1.447, 2.442)	< 0.001	0.503	1.653 (1.264, 2.163)	< 0.001
Tumor encapsulation	−0.210	0.810 (0.628, 1.046)	0.107			
Microvascular invasion	0.551	1.734 (1.343, 2.239)	< 0.001	0.356	1.428 (1.095, 1.862)	0.009
Satellite nodules	0.927	2.526 (1.813, 3.518)	< 0.001	0.399	1.490 (1.006, 2.206)	0.046
Necrosis	0.421	1.524 (1.175, 1.977)	0.002	0.044	1.045 (0.785, 1.391)	0.761
Resection margin > 1 cm	−0.041	0.960 (0.607, 1.518)	0.862			
Operation time > 200 min	0.360	1.434 (1.110, 1.852)	0.006	0.209	1.233 (0.939, 1.619)	0.132
Blood loss > 400 mL	0.635	1.887 (1.448, 2.459)	< 0.001	0.342	1.407 (1.065, 1.860)	0.016
Blood transfusion	0.424	1.528 (1.061, 2.200)	0.023	0.050	1.051 (0.701, 1.577)	0.808
Major resection	0.511	1.668 (1.291, 2.553)	< 0.001	0.057	1.059 (0.796, 1.409)	0.696

*Abbreviations*: *HBsAg* hepatitis B surface antigen, *HBeAg* hepatitis Be antigen, *HBV-DNA* hepatitis B virus DNA load, *PT* prothrombin time, *T-Bil* total bilirubin, *ALB* albumin, *ALT* alanine transaminase, *AST* aspartic aminotransferase, *GGT* γ-glutamyl transpeptadase, *AFP* α-fetoprotein, *CSPH* clinically significant portal hypertension

### Development and validation of an ANN model for predicting PHER

**Fig. 1:**
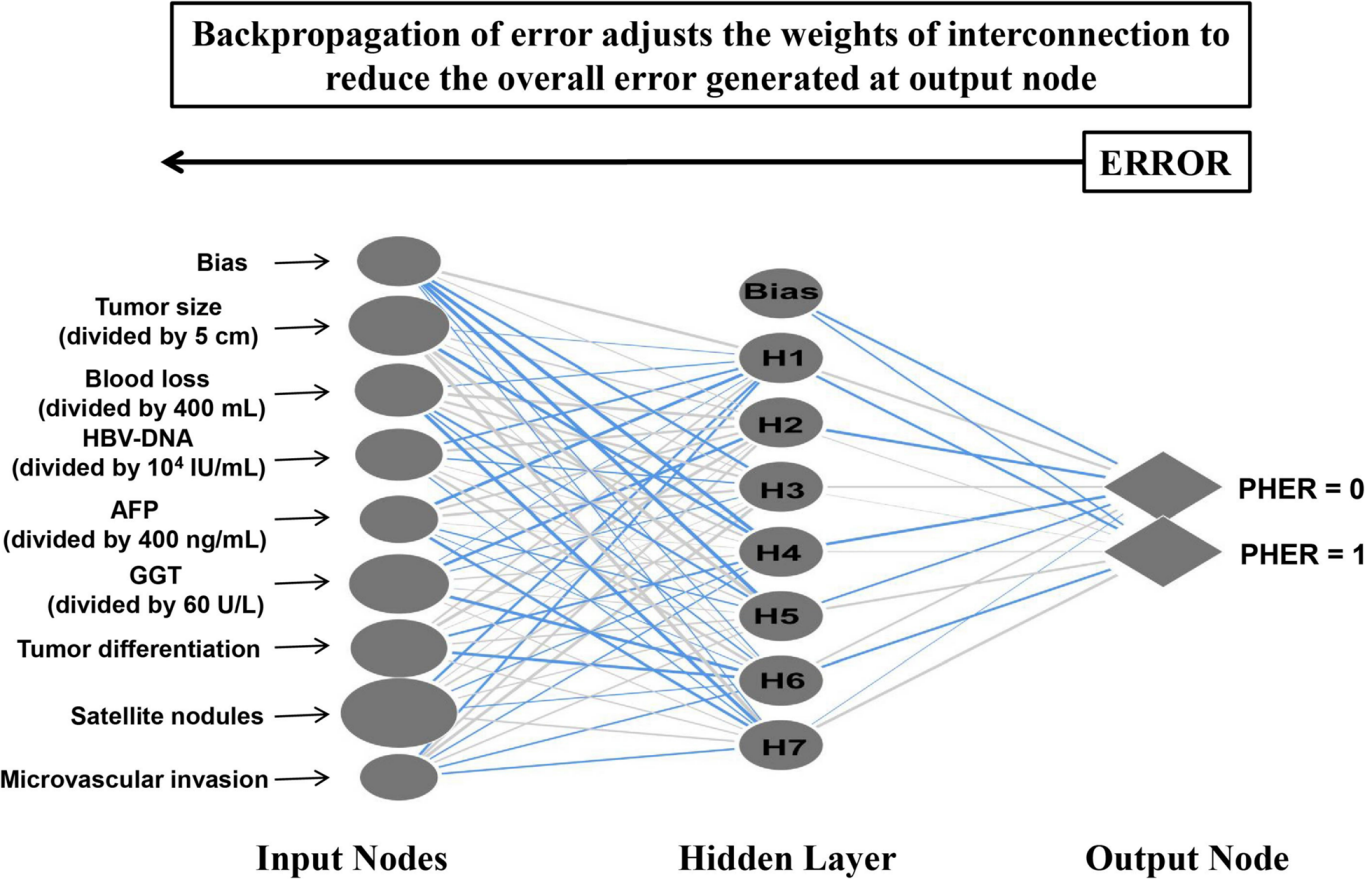
Schematic representation of the ANN model developed to predict PHER in HCC patients without macroscopic vascular invasion. The blue lines represent synaptic weights < 0, while the grey lines represent synaptic weights > 0. Abbreviations: HCC, hepatocellular carcinoma; ANN, artificial neural network; PHER, post-hepatectomy early recurrence; HBV-DNA, hepatitis B virus DNA load; GGT, γ-glutamyl transpeptadase; AFP, α-fetoprotein; MVI, microvascular invasion

**Fig. 2:**
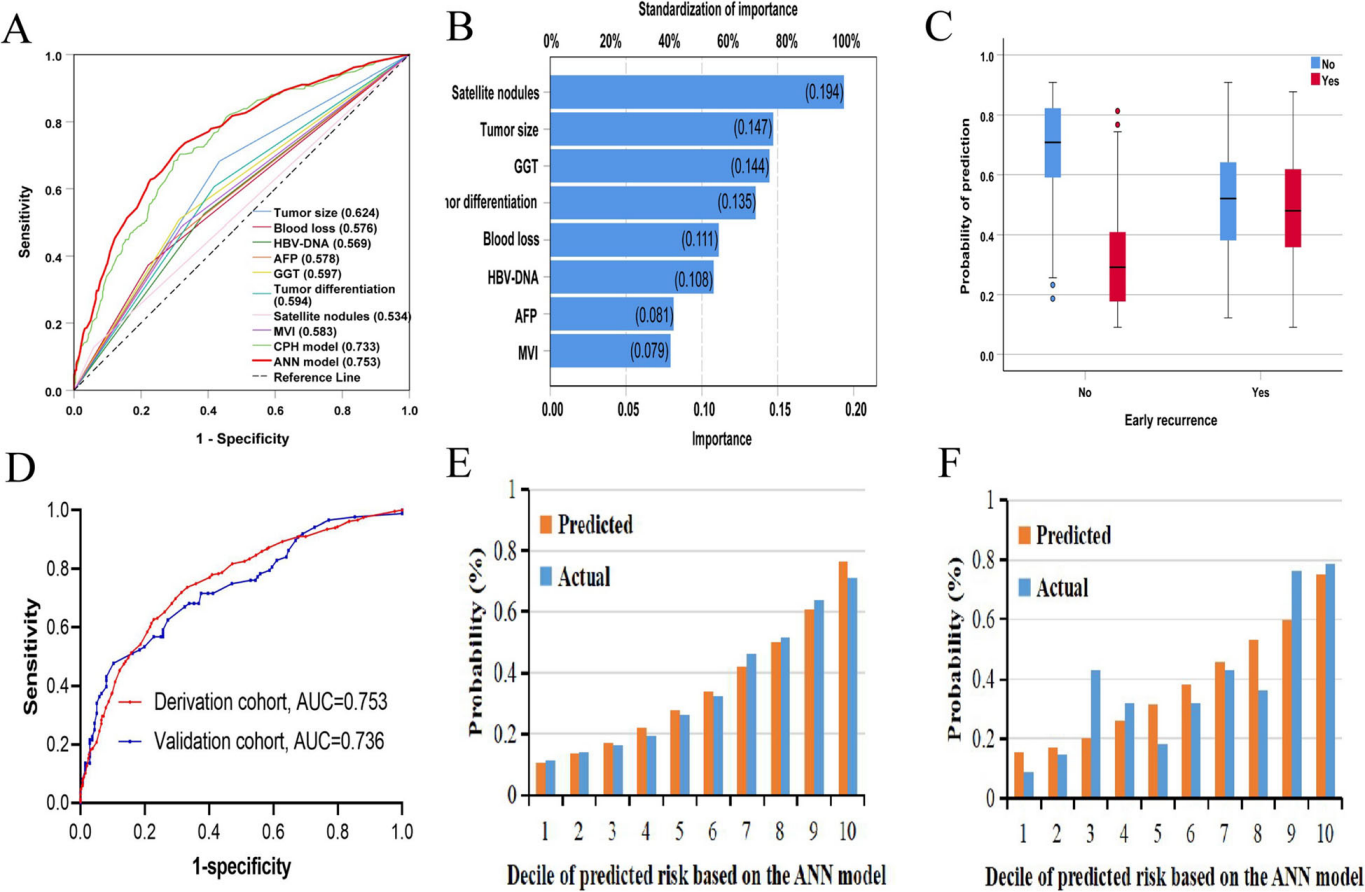
**a** ROC curves for the ANN model and the eight prognostic factors used to predict PHER. **b** The importance of the eight prognostic factors relative to the ANN model. **c** Prediction probability histograms for the ANN model in HCC patients without macroscopic vascular invasion. **d** ROC curves for the ANN model in predicting PHER in the derivation and validation cohorts. Calibration curves of the ANN model in the derivation **e** and validation **f** cohorts. The x-axis represents deciles of predicted risk, and the y-axis indicates predicted and actual probabilities of PHER. Abbreviations: ANN, artificial neural network; PHER, post-hepatectomy early recurrence; HCC, hepatocellular carcinoma; ROC, receiver operating characteristic

The CPH and ANN models were established according to the eight prognostic factors listed above (Table 2). For the ANN model (Fig. 1), the program file (Supplementary File) was downloaded onto our computer, which then automatically calculated the PHER risk in clinical applications (Supplementary Fig. 3). The subsequent ROC analysis revealed that the ANN model predicted PHER better than the CPH model (ANN: 0.753 versus CPH: 0.733, *P* < 0.05; Fig. 2a, Supplementary Table 2) as well as the eight prognostic factors individually (ANN: 0.753 versus corresponding AUCs: 0.534–0.624, *P* < 0.05 for all comparisons; Fig. 2a, Supplementary Table 2). An analysis of the importance of the eight prognostic factors revealed that the presence of satellite nodules is the most important factor in the ANN model (100%), followed by tumor size (76.0%), GGT level (74.6%), tumor differentiation (70.0%), blood loss (57.4%), HBV-DNA load (55.7%), AFP level (42.0%), and MVI (41.0%; Fig. 2b).

The prediction probability plot (Fig. 2c) indicated that the ANN model can accurately identify patients without PHER. The AUCs of the ANN model for assessing PHER risk were 0.753 (95% CI: 0.715–0.792) and 0.736 (95% CI: 0.668–0.803) in the derivation and validation cohorts, respectively (Fig. 2d). Moreover, calibration plots detected a good correlation between prediction and observation of the two cohorts (Fig. 2e and f).

### The predictive ability of the ANN model when compared to other models and staging systems

**Fig. 3:**
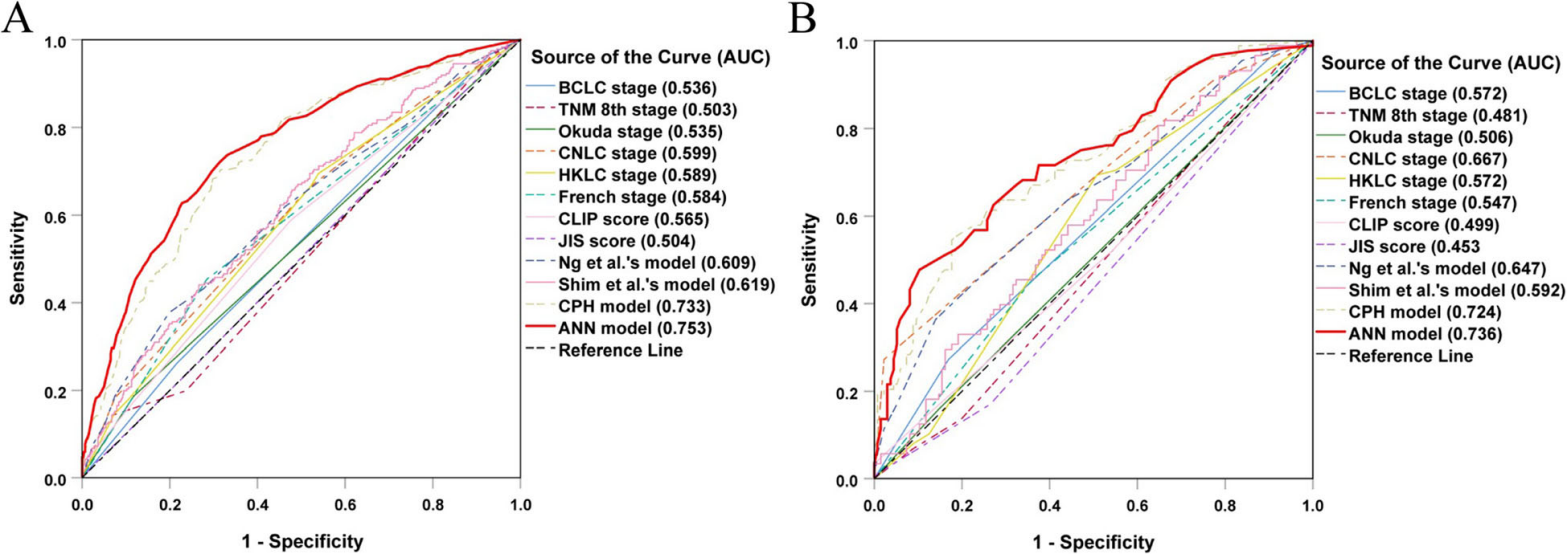
ROC curves for the ANN model and other models and staging systems in predicting PHER in the derivation **a** and validation **b** cohorts. Abbreviations: ROC, receiver operating characteristic; ANN, artificial neural network; PHER, post-hepatectomy early recurrence

**Fig. 4:**
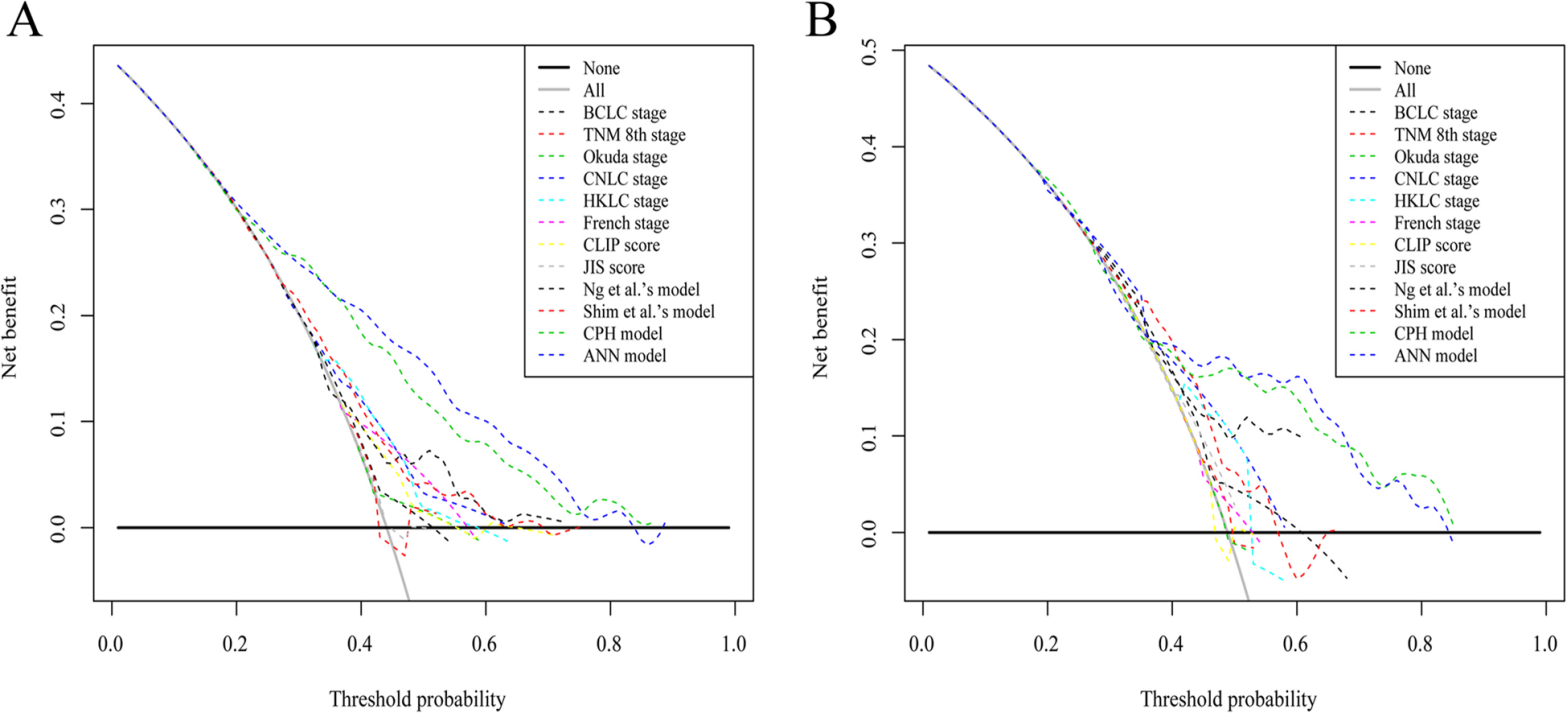
DCA plot for the ANN model and other models and staging systems in predicting PHER in the derivation **a** and validation **b** cohorts. Abbreviations: DCA, decision curve analyses; ANN, artificial neural network; PHER, post-hepatectomy early recurrence

We compared the predictive ability of the ANN model with other models and staging systems using their AUC values and net benefits. As shown in Table 3, the ROC analysis revealed that the AUC of the ANN model (0.753) was larger than all other models and staging systems (corresponding AUCs: 0.489–0.733, *P* < 0.05 for all comparisons; Fig. 3a). Moreover, the DCA plot indicated that the ANN model demonstrates better net benefit with a wider threshold probability range (Fig. 4a). Accordingly, the ANN model was superior in predicting PHER in the derivation cohort. Similar results were obtained in the validation cohort. Although the AUC values between the ANN and some models (i.e., CNLC stage and CPH model) did not significantly differ, the ANN model still had the largest AUC value and net benefit (Figs. 3 and 4b, b).

**Table 3:** The performance of the ANN model and other models and staging systems in predicting early recurrence in derivation cohort and training cohort

Staging systems / models	Training cohort			Validation cohort		
	AUC	95% CI	*P* vaule	AUC	95% CI	*P* vaule
BCLC stage	0.536	0.495–0.648	< 0.05	0.572	0.495–0.648	< 0.05
TNM^8th^ stage	0.503	0.458–0.549	< 0.05	0.481	0.405–0.558	< 0.05
Okuda stage	0.535	0.428–0.584	< 0.05	0.506	0.428–0.584	< 0.05
CNLC stage	0.599	0.593–0.741	< 0.05	0.667	0.593–0.741	> 0.05
HKLC stage	0.589	0.496–0.648	< 0.05	0.572	0.496–0.648	< 0.05
French stage	0.584	0.470–0.625	< 0.05	0.547	0.470–0.625	< 0.05
CLIP score	0.565	0.421–0.578	< 0.05	0.499	0.421–0.578	< 0.05
JIS score	0.504	0.376–0.529	< 0.05	0.453	0.376–0.529	< 0.05
Ng et al.’s model	0.609	0.573–0.721	< 0.05	0.647	0.573–0.721	< 0.05
Shim et al.’s model	0.619	0.517–0.667	< 0.05	0.592	0.517–0.667	< 0.05
CPH model	0.733	0.657–0.792	< 0.05	0.724	0.657–0.792	> 0.05
ANN model	0.753	0.668–0.803	Ref	0.736	0.668–0.803	Ref

*Abbreviations*: *BCLC* Barcelona Clinic Liver Cancer, *TNM 8th* 8th edition of TNM /AJCC, *CNLC* China Liver Cancer, *HKLC* Hong Kong Liver Cancer, *CLIP* Cancer of the Liver Italian Program, *JIS* Japan Integrated Staging, *CPH* Cox’s proportional hazards, *ANN* artificial neural network

### Risk stratification performance based on the ANN model

**Fig. 5:**
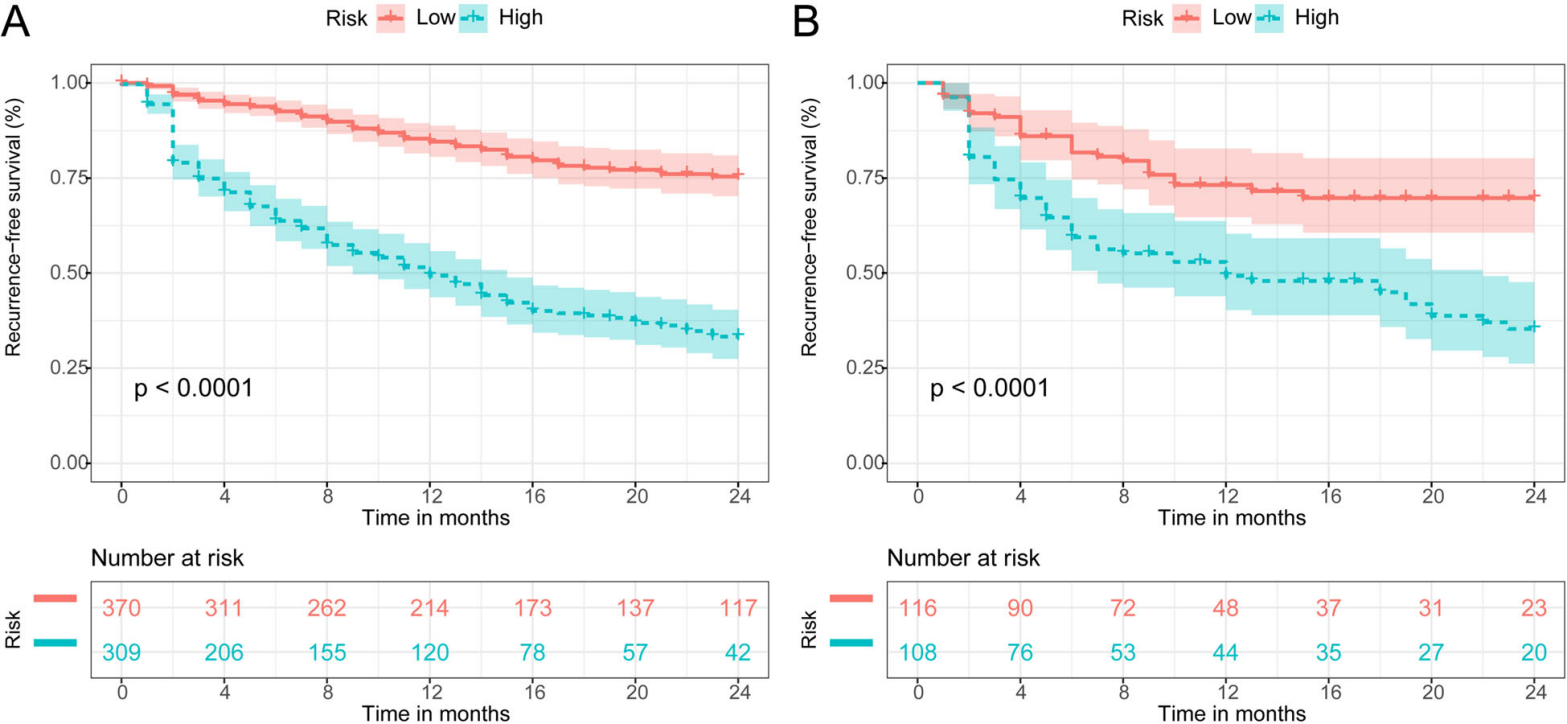
Recurrence-risk stratification of the ANN model in the derivation **a** and validation **b** cohorts. Abbreviations: ANN, artificial neural network

According to the Youden’s index, the optimal cut-off value for the ANN model to predict PHER was 0.37, and its sensitivity and specificity were 72.0 and 68.6%, respectively. Thus, we obtained a risk stratification of two groups, low risk (≤0.37) and high risk (> 0.37). Furthermore, in the derivation and validation cohorts, the RFS curves for all patients were stratified by these risk groups (Fig. 5). These results suggest that the high-risk group is closely related to poorer prognosis (*P* < 0.001 for all comparisons).

## Discussion

This retrospective study developed an ANN model to predict PHER in HCC patients without macroscopic vascular invasion. Our ANN model achieved satisfactory discriminatory and calibration capacities in both the derivation and validation cohorts. In addition, it demonstrated greater prediction capacity than a CPH model, some preexisting recurrence models, and commonly used staging systems. Finally, the ANN model stratified patients into two risk groups, highlighting the significant differences in RFS between different risk groups.

The high incidence of PHER remains a major challenge for clinicians [2–4]. Early tumor recurrence is associated with intra-hepatic metastasis, while late tumor recurrence is closely related to multi-centric oncogenesis [6, 7]. Despite similar adjuvant treatments, patients with PHER have significantly worse outcomes than patients with late recurrence [10, 11]. To prevent PHER and prolong the long-term survival of HCC patients, the effectiveness of different postoperative adjuvant treatments including RFA, interferon, TACE, and immunotherapy have been evaluated [16, 32, 33]. Ueno et al. [33] reported that TACE adjuvant can reduce PHER risk, but it cannot reduce the risk of late recurrence. In contrast, Jiang et al. [34] found that postoperative adjunctive TACE did not improve RFS and overall survival in HCC patients after curative liver resection. In fact, Ahmed et al. [35] have suggested that TACE may worsen patients’ quality of life. Systematic reviews and meta-analyses have failed to find strong evidence to support the use of these adjuvant treatments [36, 37]. These discrepancies can be attributed to the heterogeneity of patient groups in randomized and non-randomized controlled studies and their consequent differences in adjuvant treatment outcomes. Indeed, if these studies focused only on the impact of adjuvant treatments on postoperative tumor recurrence in high-risk patients, the results may have been different. Therefore, the identification of high-risk patients with PHER is clinically significant; these patients can become the focus of future clinical studies on adjuvant therapies. An accurate, reliable, and specific prediction tool based on ready-made prognostic variables can accurately identify patients at high-risk for PHER and therefore be an effective method to address this clinical problem. The prediction model may also closely relate to the monitoring of postoperative tumor recurrence. In theory, high-risk patients should adopt more aggressive and effective monitoring programs such as the use of more accurate radiological assays to detect PHER and thereby allow more timely remedial measures to be taken. For example, some centers perform preventive remedial transplants after hepatic resection in high-risk patients to prolong their long-term survival [38].

Numerous clinical and pathological prognostic factors have been identified to cause tumor recurrence following curative hepatectomy. However, few studies have been published on the establishment of accurate and effective models to predict PHER. To date, most proposed models do not focus specifically on it. These models include: the recurrence clinical risk score from Lee et al. [39], the Shanghai score from Sun et al. [40], the recurrence score for hepatitis B virus-related HCC proposed by Qin et al. [41], a RFS nomogram for AFP-negative patients created by Gan et al. [42], early recurrence after surgery for liver tumor models built by Chan et al. [5], the Hong Kong recurrent model developed by Ng et al. [8], the recurrence after curative hepatectomy score constructed by Tokumitsu [43], and some radiomics-based prognostic prediction models [44–47]. Although these models are accurate, they were created using traditional linear models such as CPH model and survival analysis. The correlation between different risk factors is multidimensional, complex, and non-linear. Therefore, correlational analyses between these factors are limited when they are applied using only traditional linear methods. An ANN model is probably more effective when multiple risk factors are involved in multidimensional and complex functions that interact with each other [12–16]. In this study, based on the eight most important prognostic factors, we developed an ANN model that performed better in predicting PHER in HCC patients without macroscopic vascular invasion than a CPH model as well as some preexisting recurrence models (Figs. 3 and 4, Table 3).

Our data revealed that HBV-DNA, GGT levels, AFP levels, tumor size, tumor differentiation, MVI, satellite nodules, and blood loss were associated with PHER (Table 2). These factors are common and readily assayed in clinical practice. Past studies have reported that the higher the HBV-DNA load, the greater the risk of low survival and tumor recurrence after hepatectomy [48–50]. High GGT levels may lead to liver dysfunction by inducing deoxyribonucleic acid instability, tumorigenesis, and cancer progression. Additionally, high GGT levels predict poorer HCC prognosis [51, 52]. High AFP levels indicate that the tumor is highly aggressive; the probability of intra-hepatic metastasis is usually greater in patients with high AFP levels [53]. Others have reported that larger tumor sizes are significantly associated with PHER [54] and that tumor differentiation, MVI, and satellite nodules relate to more severe PHER [55–57]. Finally, excessive blood loss often leads to systemic inflammatory reactions and reduces immunity, leading to increased risk of serious complications and tumor recurrence after surgery. All these parameters are easily found in medical records, thereby facilitating routine use of the ANN model relative to other models that use complex radiological variables [44–47].

The ANN model built in this paper is more accurate in predicting PHER than commonly used staging systems (Figs. 3 and 4, Table 3). The increased accuracy is likely because these systems and models contain very few parameters and try to balance risk variables by summarizing them. Accordingly, simplified models may limit PHER prediction accuracy in HCC patients. Moreover, these systems and models are linearly additive forms based on prognostic variables, and the interactions between prognostic variables cannot be accurately delineated. ANN models, in contrast, contain a wide range of predictors and manage the interactions between all prognostic factors, consequently improving their predictive power.

Currently, no clear consensus or guidelines exist on the ideal adjuvant therapy after hepatectomy. Moreover, the criteria for identifying patients at high risk for PHER remain unclear. Notably, the ANN model can be stored in a computer as a program (Supplementary File). After the clinician enters the eight prognostic factors (HBV-DNA, GGT level, AFP level, tumor size, tumor differentiation, MVI, satellite nodules, and blood loss) into the program, it will automatically and accurately calculate PHER risk (Supplementary Fig. 3). Here, our ANN model had a cut-off value of 0.37, and its sensitivity and specificity, respectively, in assessing PHER risk was 72.0 and 68.6%. All patients were then divided into high-risk and low-risk groups. The risk stratification analysis detected significant differences in RFS curves between the two risk groups (Fig. 5; *P* < 0.05 for all comparisons). As expected, patients in the high-risk group had poor RFS; however, the screening of these patients can greatly positively impact adjuvant therapy strategies. For instance, in low-risk patients, the appropriate adjuvant therapy should reduce side effects, especially in elderly patients. In contrast, high-risk patients may need to combine adjuvant treatments to obtain optimal prognoses, particularly in younger patients. A recent meta-analysis reported that in HCC patients, TACE + RFA can provide therapeutic outcomes comparable with hepatectomy but with the advantage of reduced morbidity [58]. This finding is important for clinical decision making in high-risk patients, as it is worth considering whether a patient deemed to be at high risk of PHER should undergo a hepatectomy. Accordingly, high-risk patients must be closely monitored, and appropriate treatment options must be explored. Finally, the ANN model allows the stratification of PHER risk in patients in a way more appropriate for the design of clinical trials.

Despite the promising data presented here, this study does have some limitations. First, clinicians have no mathematical formula to use directly in the ANN model, which may limit its wide-spread use. Therefore, more convenient algorithms for ANN models should be developed. Also, most of the patients in our study had HBV infections; therefore, further validation in other etiological populations is necessary. Finally, this study is retrospective and was conducted with patients from a single medical center. Thus, prospective studies using patients from several medical centers are required to verify the results obtained here.

## Conclusion

An accurate, reliable, and specific ANN model that predicts PHER risk in HCC patients without macroscopic vascular invasion was established and validated in this paper. The ANN model provides valuable data to help identify high-risk patients for future adjuvant therapy and active surveillance studies.

## Supplementary Information

**Additional file 1: Supplementary Table 1.**Staging systems of whole cohort, derivation cohort and validation cohort. **Supplementary Table 2.** The performance of the ANN model, Cox model and these eight prognostic factors individually in predicting post-hepatectomy early recurrence in derivation cohort**. Supplementary Figure 1.** Flow chart of the study design. **Supplementary Figure 2.** Recurrence-risk stratification curves of the eight prognostic factors in the derivation cohort. (A) HBV-DNA, (B) GGT) level, (C) AFP level, (D) tumor size), (E) tumor differentiation, (F) MVI, (G) satellite nodules, (H) blood loss. Abbreviations: HBV-DNA, Hepatitis B virus deoxyribonucleic acid load; GGT, γ-glutamyl transpeptidase; AFP, α-fetoprotein; MVI; microvascular invasion. **Supplementary Figure 3.** Screenshot of the SPSS software-based ANN model to predict PHER risk. First, SPSS is opened, and the values of the eight prognostic factors (HBV-DNA load, GGT level, AFP level, tumor size, tumor differentiation, MVI, satellite nodules, and blood loss) are entered into the program. Next, the Scoring Wizard on the toolbar is found, and the ANN model file is selected. The program will automatically calculate PHER risk. For ex ample, in an HCC patient with high levels of HBV-DNA, GGT, and AFP; large tumor size; poor tumor differentiation; high blood loss; and presence of MVI and satellite nodules, the program calculated a predicted PHER risk of 0.84. This patient was classified in the high-risk group based on the risk stratification. Abbreviations: HCC, hepatocellular carcinoma; ANN, artificial neural network; PHER, post-hepatectomy early recurrence; HBV-DNA, hepatitis B virus DNA load; GGT, γ-glutamyl transpeptadase; AFP, α-fetoprotein; MVI, microvascular invasion.

## Data Availability

The data used or analyzed during this study are included in this article and available from the corresponding author upon reasonable request.

## Abbreviations

HCC: Hepatocellular carcinoma
PHER: Post-hepatectomy early recurrence
ANN: Artificial neural network
HBsAg: Hepatitis B surface antigen
HBV-DNA: Hepatitis B virus DNA load
AST: Aspartate aminotransferase
GGT: γ-glutamyl transpeptadase
PT: Prothrombin time
AFP: α-fetoprotein
MVI: Microvascular invasion
BCLC: Barcelona Clinic Liver Cancer
TNM 8th: 8th edition of TNM /AJCC
CNLC: China Liver Cancer
HKLC: Hong Kong Liver Cancer
CLIP: Cancer of the Liver Italian Program
JIS: Japan Integrated Staging
RFA: Radiofrequency ablation
TACE: Transcatheter arterial chemoembolization
ROC: Receiver-operating characteristic
AUC: Area under the ROC curve
DCA: Decision curve analyses
RFS: Recurrence-free survival
