# Supplementary material for: Artificial neural network model to predict post-hepatectomy early recurrence of hepatocellular carcinoma without macroscopic vascular invasion
Source: BMC Cancer. 2021 Mar 16;21:283. doi: 10.1186/s12885-021-07969-4 (PMC7962237; doi:10.1186/s12885-021-07969-4)

**Supplemental contents to:**

**Artificial neural network model to predict post-hepatectomy early recurrence of hepatocellular carcinoma without macroscopic vascular invasion**

*Rong-yun Mai, Jie Zeng, Wei-da Meng, Hua-ze Lu, Rong Liang, Yan Lin, Guo-bin Wu, Le-qun Li, Liang Ma , Jia-zhou Ye and Tao Bai*

**Table of Contents**

**Supplementary Table 1**.Staging systems of whole cohort, derivation cohort and validation cohort 2

**Supplementary Table 2.** The performance of the ANN model, Cox model and these eight prognostic factors individually in predicting post-hepatectomy early recurrence in derivation cohort. 3

**Supplementary Figure 1.** Flow chart of study design. 4

**Supplementary Figure 2.** Recurrence-risk stratification of the eight prognostic factors in derivation cohort. 5

**Supplementary Figure 3.** Screenshot of the SPSS software-based ANN model to predict the risk of PHER. 6

Supplementary Table 1.Staging systems of whole cohort, derivation cohort and validation cohort.

| Staging systems | Whole (n=903) | Derivation (n=679) | Validation (n=224) | *P* value |
| --- | --- | --- | --- | --- |
| BCLC stage |  |  |  | 0.776 |
| 0 | 52 (5.8) | 39 (5.7) | 13 (5.8) |  |
| A | 646 (71.5) | 482 (71.0) | 164 (73.2) |  |
| B | 205 (22.7) | 158 (23.3) | 47 (21.0) |  |
| Okuda grade |  |  |  | 0.772 |
| I | 773 (85.6) | 583 (85.9) | 190 (84.8) |  |
| II | 129 (14.3) | 95 (14.0) | 34 (15.2) |  |
| III | 1 (0.1) | 1 (0.1) | 0 (0) |  |
| TNM 8th grade |  |  |  | 0.430 |
| Ia | 47 (5.2) | 36 (5.3) | 11 (4.9) |  |
| Ib | 665 (73.6) | 491 (72.3) | 174 (77.7) |  |
| II | 107 (11.8) | 86 (12.7) | 21 (9.4) |  |
| IIIa | 84 (9.3) | 66 (9.7) | 18 (8.0) |  |
| CNLC grade |  |  |  | 0.886 |
| Ia | 172 (19.0) | 133 (19.6) | 39 (17.4) |  |
| Ib | 213 (23.6) | 158 (23.3) | 55 (24.6) |  |
| IIa | 415 (46.0) | 312 (45.9) | 103 (46.0) |  |
| IIb | 103 (11.4) | 76 (11.2) | 27 (12.1) |  |
| French grade |  |  |  | 0.142 |
| A | 581 (64.3) | 446 (65.7) | 135 (60.3) |  |
| B | 322 (35.7) | 233 (34.3) | 89 (39.7) |  |
| HKLC grade |  |  |  | 0.842 |
| I | 363 (40.2) | 275 (40.6) | 87 (38.8) |  |
| IIa | 22 (2.4) | 15 (2.2) | 7 (3.1) |  |
| IIb | 401 (44.4) | 297 (43.7) | 104 (46.4) |  |
| IIIa | 32 (3.5) | 25 (3.7) | 7 (3.1) |  |
| IIIb | 85 (9.4) | 66 (9.7) | 19 (8.5) |  |
| CLIP score |  |  |  | 0.499 |
| 0 | 433 (48.0 | 329 (48.5) | 104 (46.4) |  |
| 1 | 350 (38.8) | 257 (37.8) | 93 (41.5) |  |
| 2 | 94 (10.4) | 70 (10.3) | 24 (10.7) |  |
| 3 | 24 (2.7) | 21 (3.1) | 3 (1.3) |  |
| 4 | 2 (0.2) | 2 (0.3) | 0 (0) |  |
| JS score |  |  |  | 0.198 |
| 0 | 16 (1.8) | 14 (2.1) | 2 (0.9) |  |
| 1 | 657 (72.8) | 486 (71.6) | 171 (76.3) |  |
| 2 | 209 (23.1) | 160 (23.6) | 49 (21.9) |  |
| 3 | 21 (2.3) | 19 (2.8) | 2 (0.9) |  |

Abbreviations: BCLC, Barcelona Clinic Liver Cancer; TNM 8^th^, 8th edition of TNM /AJCC; CNLC, China Liver Cancer; HKLC, Hong Kong Liver Cancer; CLIP, Cancer of the Liver Italian Program; JIS,Japan Integrated Staging.

Supplementary Table 2. The performance of the ANN model and these eight prognostic factors individually in predicting post-hepatectomy early recurrence in derivation cohort.

| Risk factor | AUC | 95 CI% | *P* value |
| --- | --- | --- | --- |
| Tumor size | 0.624 | 0.581 - 0.668 | <0.05 |
| Blood loss | 0.576 | 0.530 - 0.622 | <0.05 |
| HBV-DNA | 0.569 | 0.523 - 0.614 | <0.05 |
| AFP | 0.578 | 0.533 - 0.533 | <0.05 |
| GGT | 0.597 | 0.552 - 0.643 | <0.05 |
| Tumor differentiation | 0.594 | 0.549 - 0.639 | <0.05 |
| Satellite nodules | 0.534 | 0.488 - 0.581 | <0.05 |
| MVI | 0.583 | 0.538 - 0.629 | <0.05 |
| CPH model | 0.733 | 0.657 - 0.792 | <0.05 |
| ANN model | 0.753 | 0.715 - 0.792 | Ref |

Abbreviations: HBV-DNA, hepatitis B virus DNA load; AFP, α-fetoprotein; GGT, γ-glutamyl transpeptadase; MVI, microvascular invasion; ANN, artificial neural network.

**Supplementary Figure 1**. Flow chart of study design.


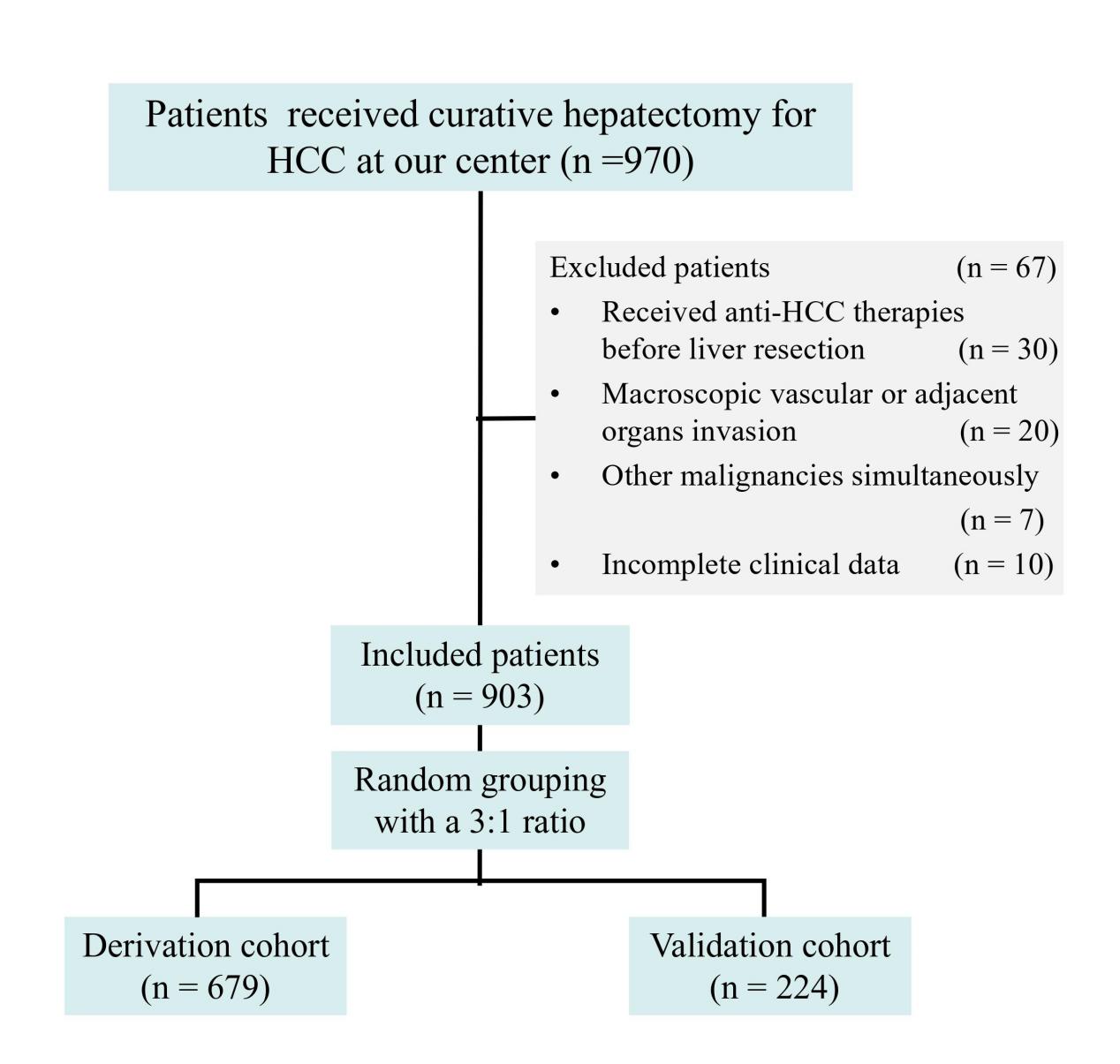


**Supplementary Figure 2**: Recurrence-risk stratification of the eight prognostic factors in derivation cohort. (A) HBV-DNA; (B) GGT; (C) AFP; (D) Tumor size; (E) Tumor differentiation; (F) MVI; (G) satellite nodules; (H) blood loss. Abbreviations: HBV-DNA, hepatitis B virus DNA load; GGT, γ-glutamyl transpeptadase; AFP, α-fetoprotein; MVI; microvascular invasion.


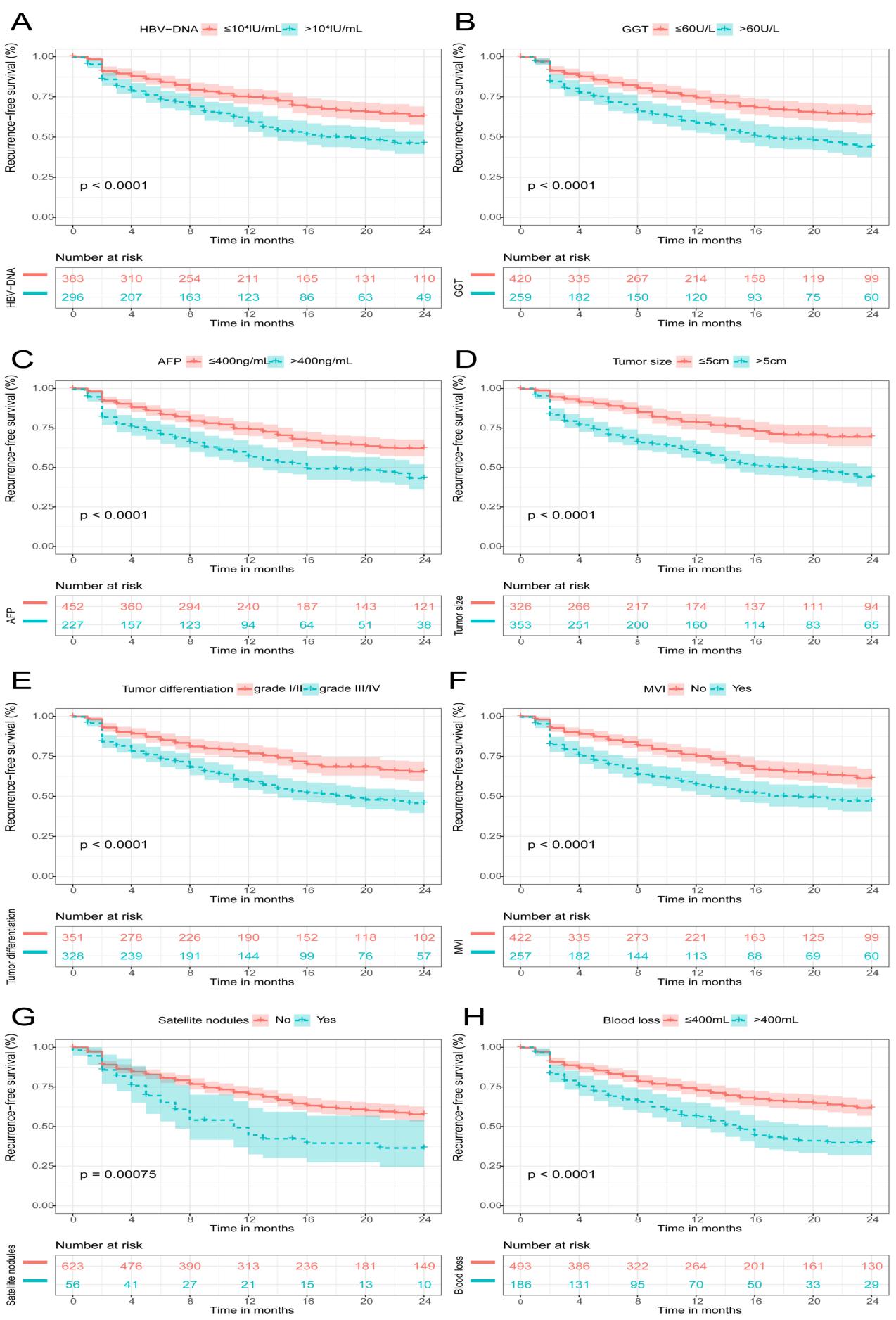


**Supplementary Figure 3.** Screenshot of the SPSS software-based ANN model to predict the risk of PHER. First open the SPSS software, enter the data of these eight prognostic factors (HBV-DNA, GGT, AFP, tumor size, tumor differentiation, MVI, satellite nodules and blood loss) into the program, then find the Scoring Wizard on the toolbar, and select our ANN model file. Finally, the computer will automatically calculate the risk of PHER. For example, when a HCC patient has high levels of HBV-DNA, GGT and AFP, large tumor size, poor tumor differentiation, high blood loss, and presence of MVI and satellite nodules, the computer calculates that the predicted risk of PHER will be 0.84. According to the risk stratification, this patient was classified as the high-risk group. Abbreviations: HCC, hepatocellular carcinoma; ANN, artificial neural network; PHER, post-hepatectomy early recurrence; HBV-DNA, hepatitis B virus DNA load; GGT, γ-glutamyl transpeptadase; AFP, α-fetoprotein; MVI, microvascular invasion.


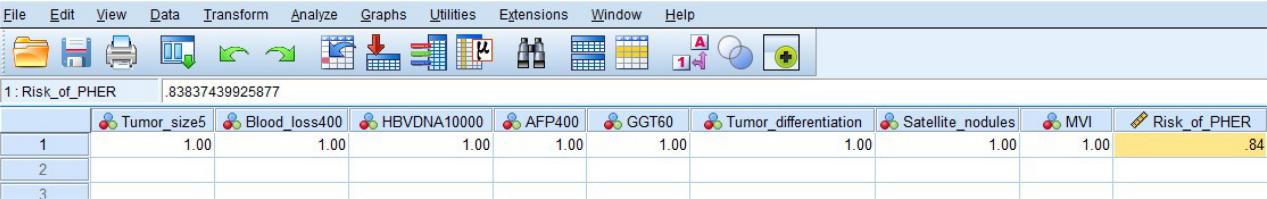

Supplement: Supplementary file 1 — Additional file 1: Supplementary Table 1.Staging systems of whole cohort, derivation cohort and validation cohort. Supplementary Table 2. The performance of the ANN model, Cox model and these eight prognostic factors individually in predicting post-hepatectomy early recurrence in derivation cohort. Supplementary Figure 1. Flow chart of the study design. Supplementary Figure 2. Recurrence-risk stratification curves of the eight prognostic factors in the derivation cohort. (A) HBV-DNA, (B) GGT) level, (C) AFP level, (D) tumor size), (E) tumor differentiation, (F) MVI, (G) satellite nodules, (H) blood loss. Abbreviations: HBV-DNA, Hepatitis B virus deoxyribonucleic acid load; GGT, γ-glutamyl transpeptidase; AFP, α-fetoprotein; MVI; microvascular invasion. Supplementary Figure 3. Screenshot of the SPSS software-based ANN model to predict PHER risk. First, SPSS is opened, and the values of the eight prognostic factors (HBV-DNA load, GGT level, AFP level, tumor size, tumor differentiation, MVI, satellite nodules, and blood loss) are entered into the program. Next, the Scoring Wizard on the toolbar is found, and the ANN model file is selected. The program will automatically calculate PHER risk. For ex ample, in an HCC patient with high levels of HBV-DNA, GGT, and AFP; large tumor size; poor tumor differentiation; high blood loss; and presence of MVI and satellite nodules, the program calculated a predicted PHER risk of 0.84. This patient was classified in the high-risk group based on the risk stratification. Abbreviations: HCC, hepatocellular carcinoma; ANN, artificial neural network; PHER, post-hepatectomy early recurrence; HBV-DNA, hepatitis B virus DNA load; GGT, γ-glutamyl transpeptadase; AFP, α-fetoprotein; MVI, microvascular invasion. [file 12885_2021_7969_MOESM1_ESM.zip › Supplementary MaterialR2.docx]
